# Supplementary figures and images for: Four tyrosine residues of the rice immune receptor XA21 are not required for interaction with the co-receptor OsSERK2 or resistance to Xanthomonas oryzae pv. oryzae
Source: PeerJ. 2018 Dec 11;6:e6074. doi: 10.7717/peerj.6074 (PMC6294051; doi:10.7717/peerj.6074)

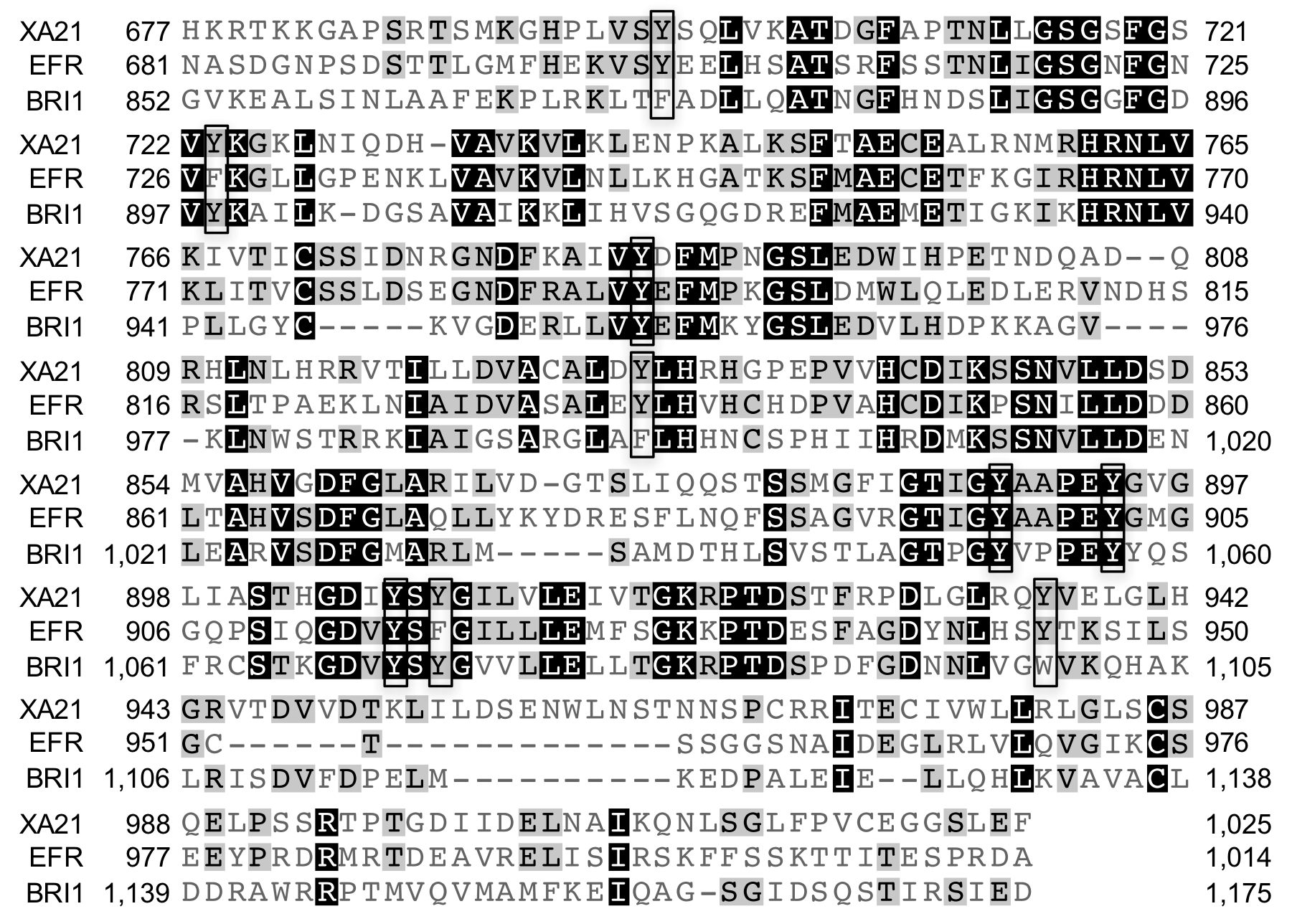

Supplement: Supplemental Information 1 — XA21JK (AA 677–AA 1,025) was used as the template to align EFR and BRI1. The amino acid position corresponds to the full length protein. The amino acids conserved between all three proteins are indicated in black. Conservation between two of the three proteins is indicated in grey. The positions of XA21 tyrosine residues are boxed. [file peerj-06-6074-s001.png]

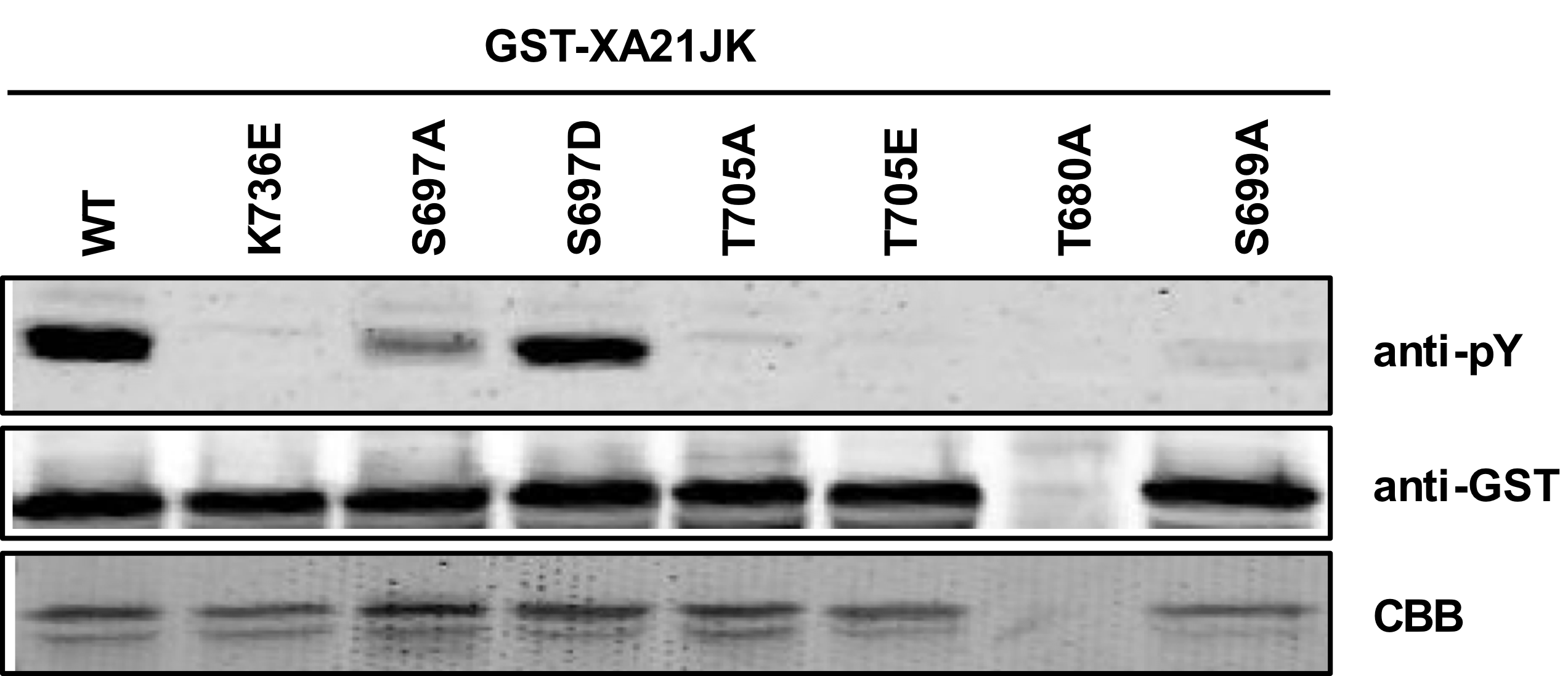

Supplement: Supplemental Information 2 — Immunoblot of GST-XA21JK variants (S697A, S697D, T705A, T705E, T680A, S699A). Anti-pY antibodies were used to identify XA21JK variants with reduced tyrosine autophosphorylation capability. Equal loading of proteins was confirmed by immunoblotting with an anti-GST antibody and Coomassie Brilliant Blue (CBB) staining of the membrane. T680A failed to express properly, as indicated by no CBB staining. [file peerj-06-6074-s002.png]

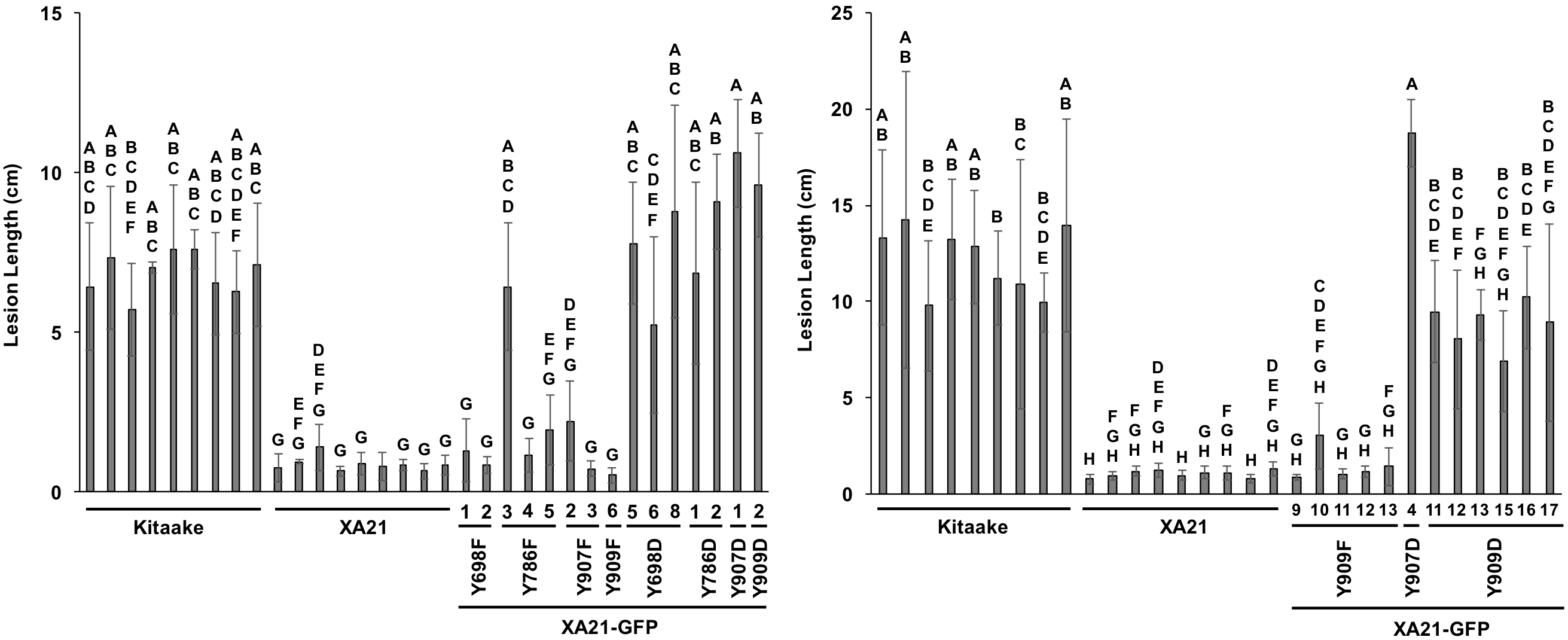

Supplement: Supplemental Information 3 — Lesion length of Kitaake, XA21 (progeny of homozygous line 7A-8), and T0 generation XA21-GFP mutant plants 14 days after inoculation with Xoo. Bars indicate the mean lesion length and standard deviation of a single plant with two to seven leaves inoculated with Xoo OD = 0.5. Different letters indicate a significant difference in lesion length (P < 0.05, Kruskal-Wallis test, Dunn’s post-hoc test with Benjamini–Hochberg correction). Shown are all transgenic lines with confirmed transgene expression by immunoblotting with anti-GFP antibodies. The left and right panels were inoculated on separate dates. [file peerj-06-6074-s003.png]

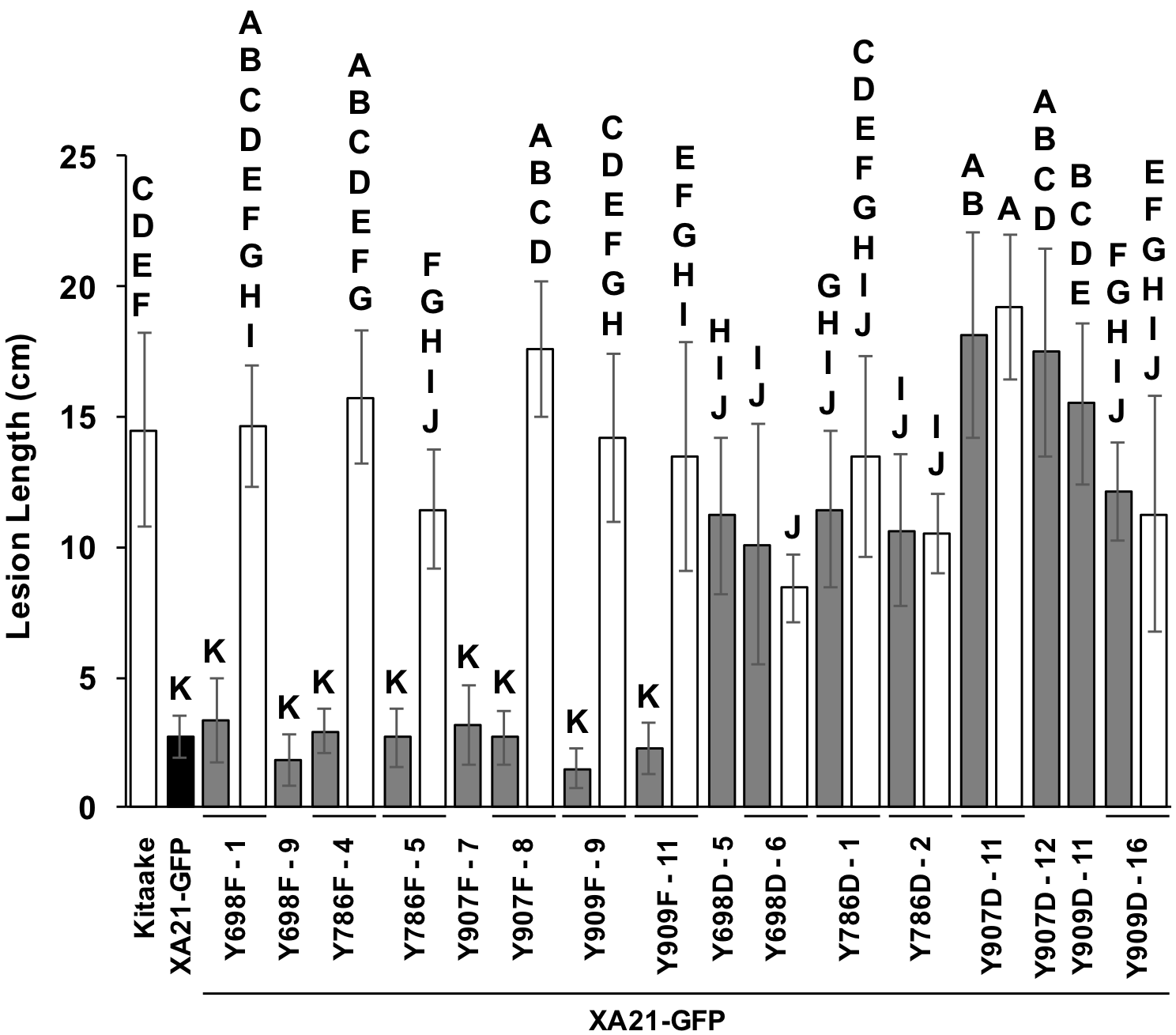

Supplement: Supplemental Information 4 — Lesion length of Kitaake, XA21-GFP, and two independently transformed T1 generation XA21-GFP variants per tyrosine substitution. Measurements were taken 14 days after inoculation with Xoo OD = 0.5. Bars indicate the mean lesion length and standard deviation of one to eight plants with four to seven leaves. Different letters indicate a significant difference in lesion length (P < 0.05, Kruskal-Wallis test, Dunn’s post-hoc test with Benjamini–Hochberg correction). Gray bars indicate the presence of the XA21-GFP construct. White bars indicate null-segregants. This experiment was performed 3 times with similar results. [file peerj-06-6074-s004.png]

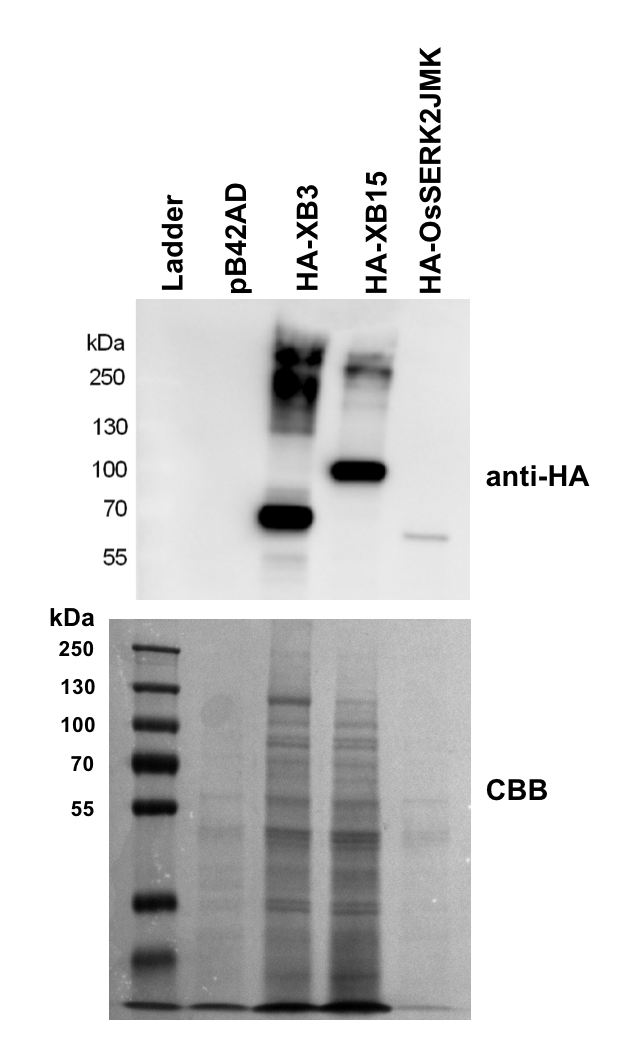

Supplement: Supplemental Information 6 [file peerj-06-6074-s006.zip › Fig7B_Raw.png]

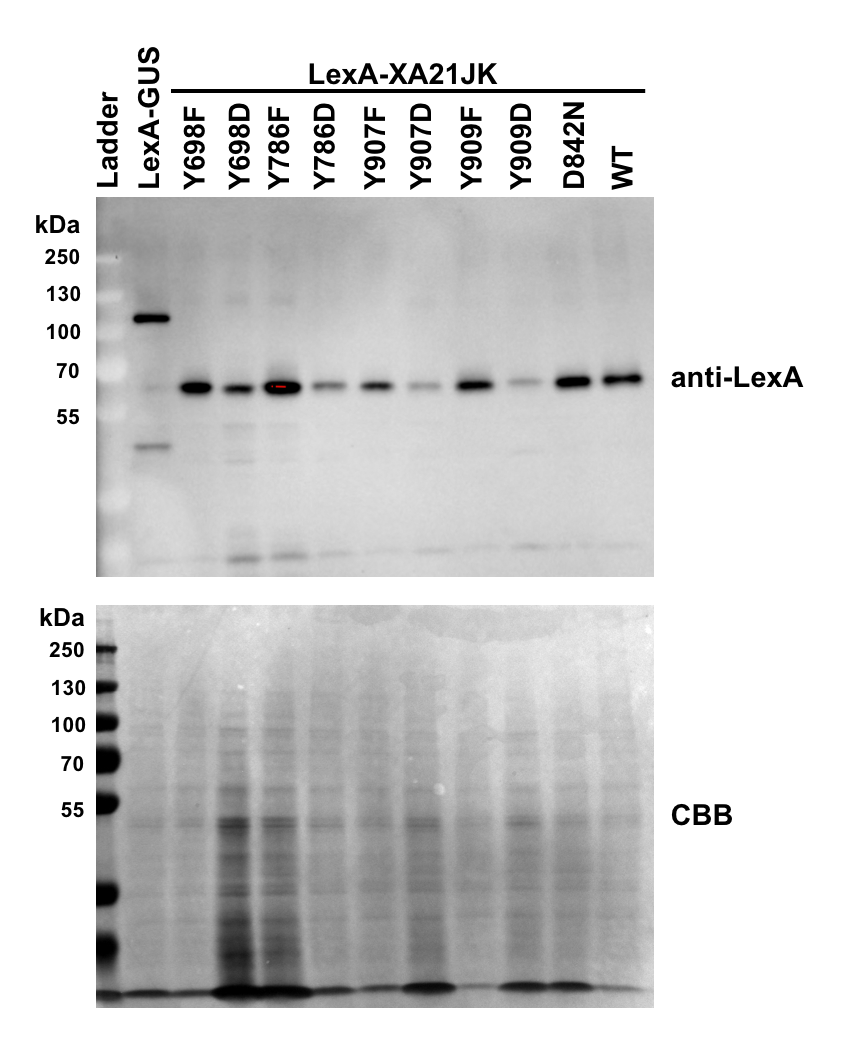

Supplement: Supplemental Information 6 [file peerj-06-6074-s006.zip › Fig7C_Raw.png]

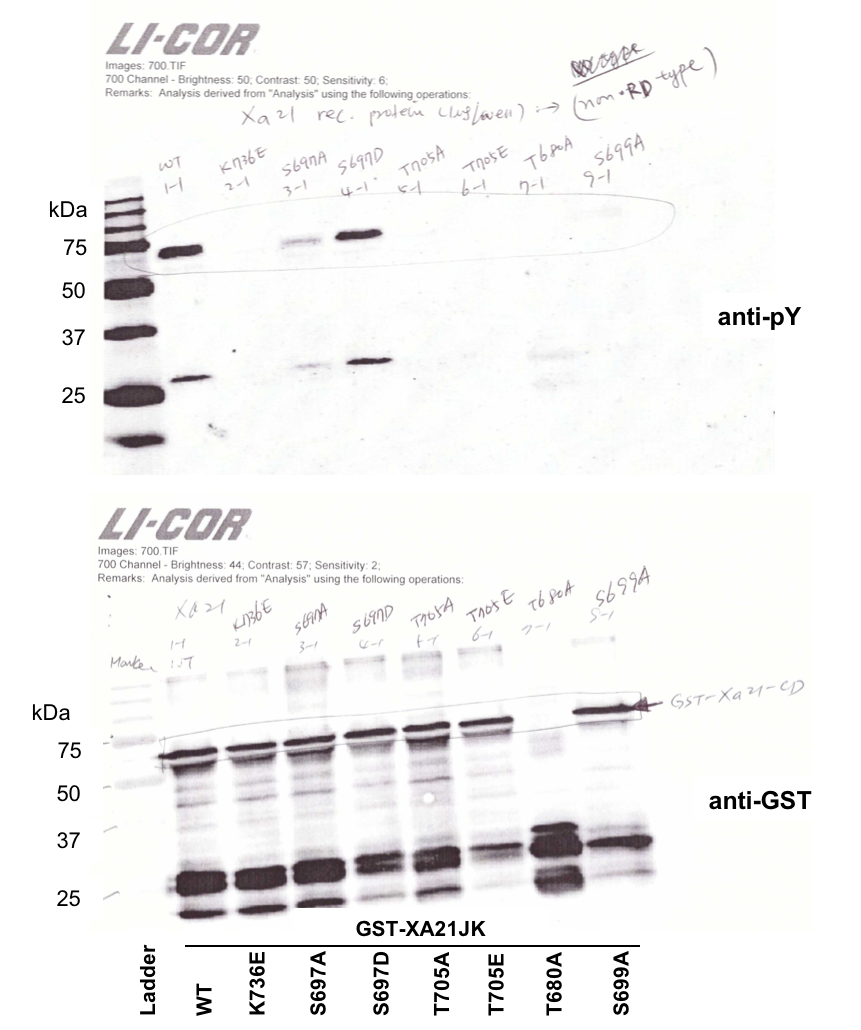

Supplement: Supplemental Information 6 [file peerj-06-6074-s006.zip › SuppFig2_Raw.png]

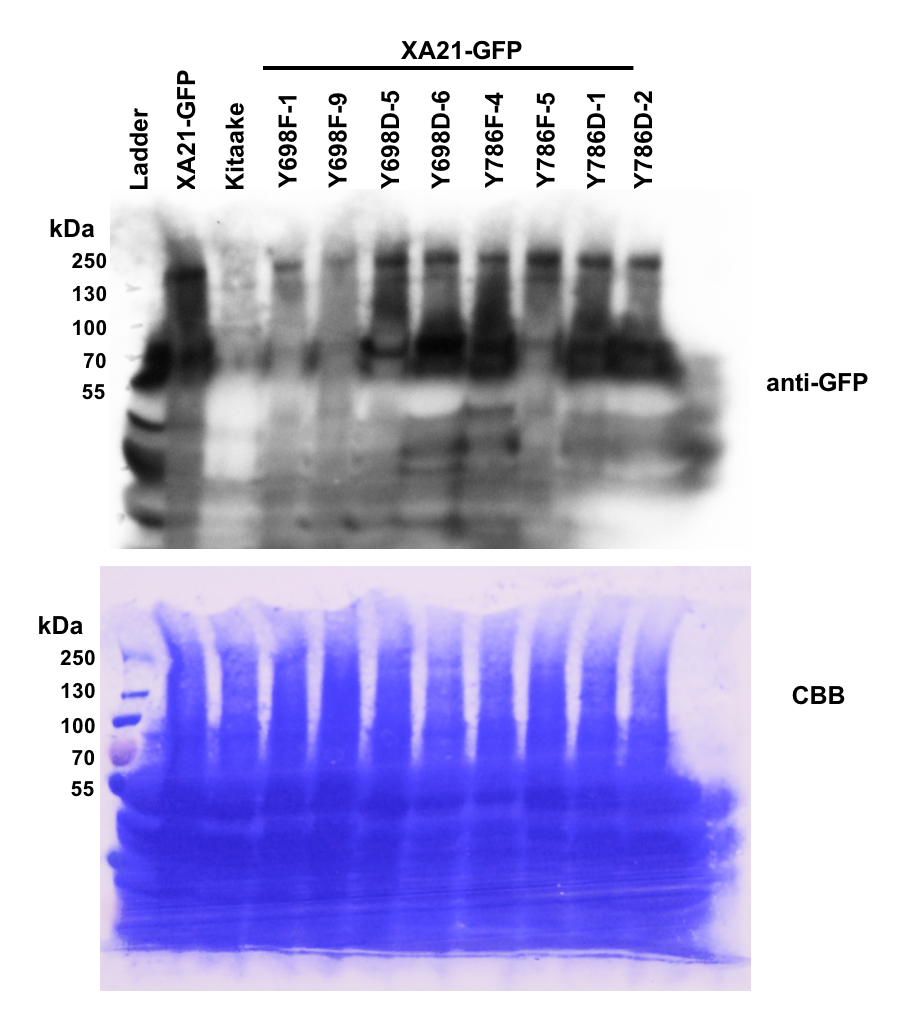

Supplement: Supplemental Information 6 [file peerj-06-6074-s006.zip › Fig4L_Raw.png]

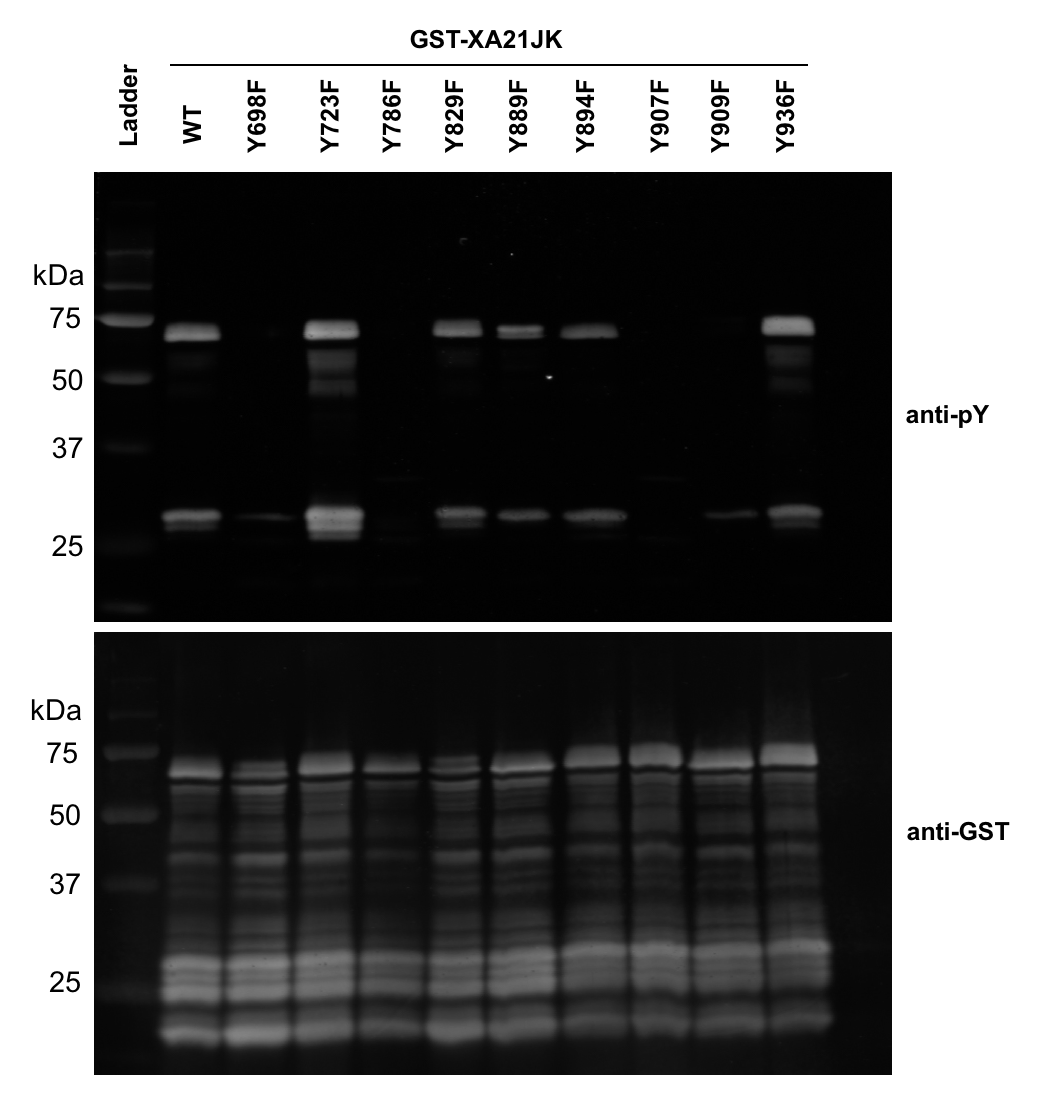

Supplement: Supplemental Information 6 [file peerj-06-6074-s006.zip › Fig1C_Raw.png]

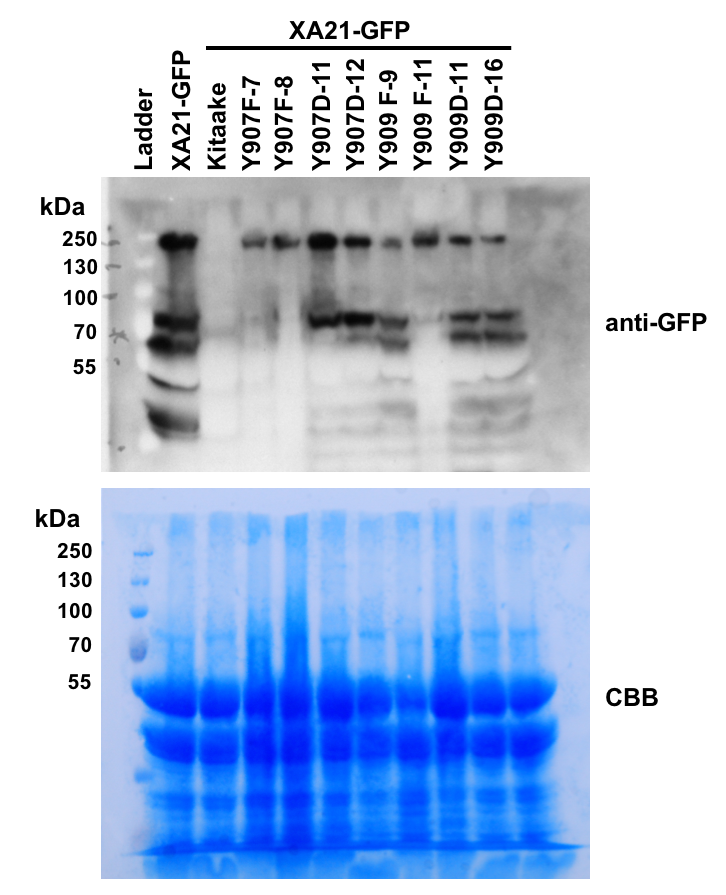

Supplement: Supplemental Information 6 [file peerj-06-6074-s006.zip › Fig4R_Raw.png]

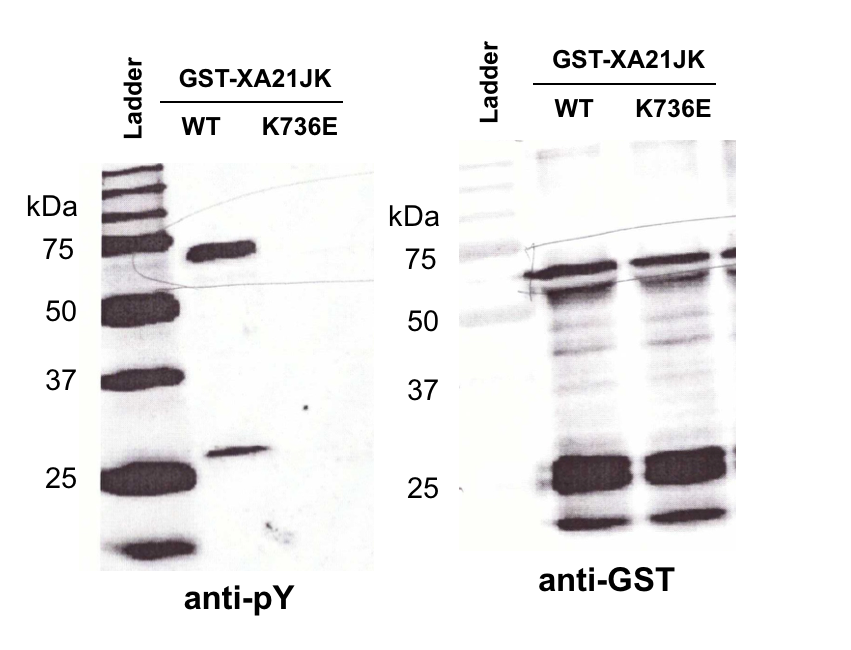

Supplement: Supplemental Information 6 [file peerj-06-6074-s006.zip › Fig1B_Raw.png]

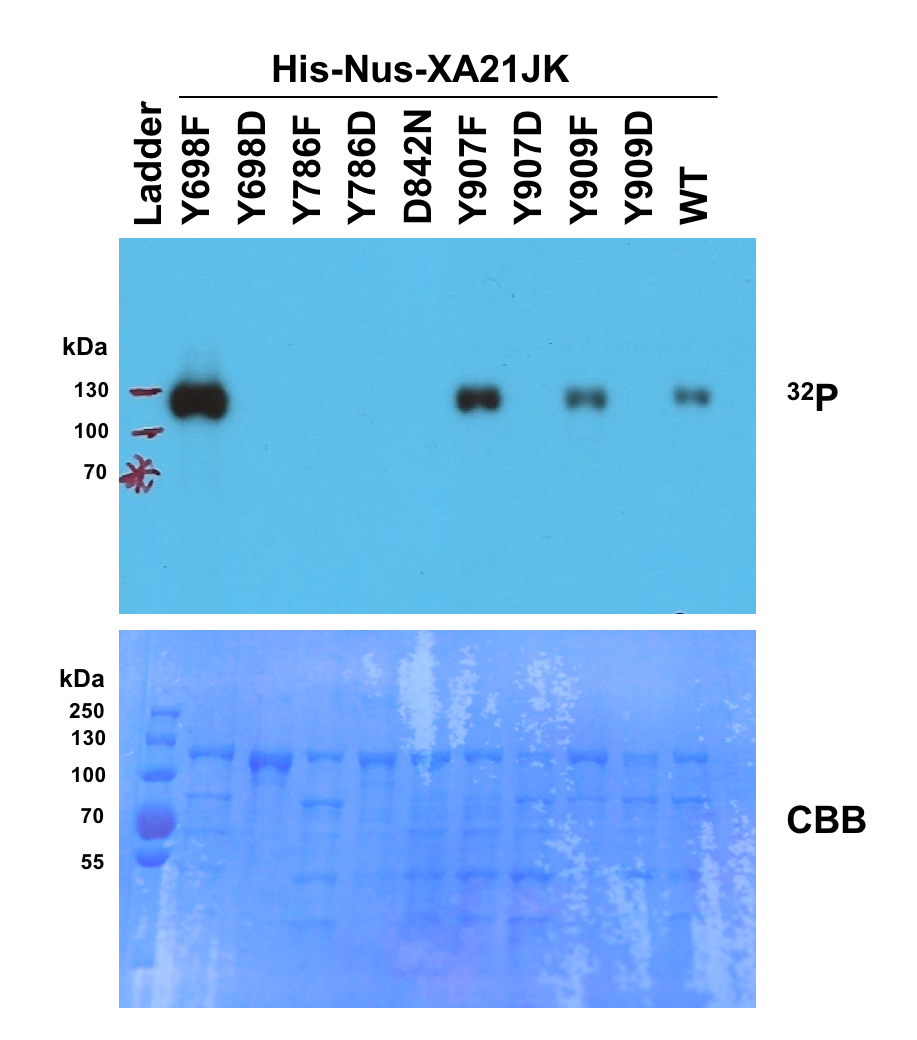

Supplement: Supplemental Information 6 [file peerj-06-6074-s006.zip › Fig2_Raw.png]
