# Supplementary material for: Four tyrosine residues of the rice immune receptor XA21 are not required for interaction with the co-receptor OsSERK2 or resistance to Xanthomonas oryzae pv. oryzae
Source: PeerJ. 2018 Dec 11;6:e6074. doi: 10.7717/peerj.6074 (PMC6294051; doi:10.7717/peerj.6074)
Supplement: Supplementary file 2 [file peerj-06-6074-s005.docx]

XA21Y907D-F	Tyrosine SDM	GCATCAACGCATGGAGATATTGACAGCTATGGAATTCTAGTGC
XA21Y907D-R		GCACTAGAATTCCATAGCTGTCAATATCTCCATGCGTTGATGC
XA21Y909D-F	Tyrosine SDM	GCATGGAGATATTTACAGCGACGGAATTCTAGTGCTGG
XA21Y909D-R		CCAGCACTAGAATTCCGTCGCTGTAAATATCTCCATGC
Ubi-pro-5’	PCR of	TTGTCGATGCTCACCCTGTTGTTT
XA21-Seq2	XA21-GFP	ACCACCTAGCTTGTTTTCTCTGAC
OsUbq-F	qPCR of	GTGGCCAGTAAGTCCTCAGC
OsUbq-R	Os06g46770	ACAATGAAACGGGACACGAC
PR10b-F	qPCR of	TGTGGAAGGTCTGCTTGGAC
PR10b-R	Os12g36850	CCTTTAGCACGTGAGTTGCG
M2-F	qPCR of	AAATGATTTGGGACCAGTCG
M2-R	Os04g10010	GATGGAATGTCCTCGCAAAC
KO5-F	qPCR of	GCTGGCTTCCAAACAAGAGC
KO5-R	Os06g37224	GCCTCTTGATCAACGCGTTC
